# Clinical epidemiology and mortality on patients with acute respiratory distress syndrome (ARDS) in Vietnam

**DOI:** 10.1371/journal.pone.0221114

**Published:** 2019-08-15

**Authors:** Luong Quoc Chinh, Toshie Manabe, Do Ngoc Son, Nguyen Van Chi, Yuji Fujikura, Nguyen Gia Binh, Dao Xuan Co, Dang Quoc Tuan, Mai Duy Ton, Khuong Quoc Dai, Pham The Thach, Hiroyuki Nagase, Koichiro Kudo, Dat Anh Nguyen

**Affiliations:** 1 Bach Mai Hospital, Emergency Department, Hanoi, Vietnam; 2 Jichi Medical University, Center of Community Medicine, Tochigi, Japan; 3 National Defense Medical College, Department of Internal Medicine, Saitama, Japan; 4 National Defense Medical College Hospital, Department of Medical Risk Management and Infection Control, Saitama, Japan; 5 Bach Mai Hospital, Intensive Care Unit, Hanoi, Vietnam; 6 Hanoi Medical University, Department of Emergency and Critical Care Medicine, Hanoi, Vietnam; 7 Teikyo University School of Medicine, Department of Respiratory Medicine, Tokyo, Japan; 8 Yurin Hospital, Tokyo, Japan; 9 Waseda University Regional and Inter-Regional Studies, Tokyo, Japan; San Gerardo Hospital, ITALY

## Abstract

**Background:**

The clinical epidemiology and disease prognosis in patients with acute respiratory distress syndrome (ARDS) have not yet been fully elucidated in Vietnam.

**Methods:**

We conducted a retrospective observational study at a national tertiary hospital in Hanoi, Vietnam. Participants were adult patients (age ≥18 years) who were admitted and diagnosed with ARDS during 2015–2017. Data on patients’ general and clinical conditions, radiographic findings, ventilator settings, gas exchange, and treatment methods were collected and compared between survivors and non-survivors. Risk factors for mortality were assessed using logistic regression analysis.

**Results:**

Among 126 eligible patients with ARDS admitted to the central tertiary hospital in Vietnam, we observed high mortality (57.1%). Of the total patients, 91.3% were transferred from local hospitals with a diagnosis of severe pneumonia and then diagnosed with ARDS at the central hospital. At the time of admission, 53.2% of patients had severe ARDS, 37.3% had moderate ARDS, and 9.5% had mild ARDS. The mean (standard deviation) sequential organ failure assessment (SOFA) score was 9.5 (3.4) in non-survivors and 7.4 (3.4) in survivors (*p* = 0.002). Although there was no significant difference in PaO_2_/FiO_2_ on admission between non-survivors and survivors, that on day 3 after admission was significantly different (*p* = 0.002). Logistic regression revealed that PaO_2_/FiO_2_ on day 3 [odds ratio (OR), 1.010; 95% confidence interval (CI), 1.003–1.017], length of stay in a local hospital before admission to the central hospital (OR, 1.122; 95% CI, 1.042–1.210) due to stable condition, and SOFA score on Day 1 (OR, 0.842; 95% CI, 0.708–1.002) were independent factors in patient survival.

**Conclusions:**

Patients with ARDS admitted the central tertiary hospital had severe illness and high mortality. Most patients were transferred from local hospitals. Improvements in human, medical, and sociological resources in local will contribute to reducing the mortality of ARDS in Vietnam.

## Introduction

Acute respiratory distress syndrome (ARDS) is a type of acute diffuse lung injury characterized by an inciting inflammation event followed by hypoxemic respiratory failure [1]. Despite advances in the care of critically ill patients, the mortality of ARDS remains high. A large observational study (LUNG SAFE) examined a sample of 459 intensive care units (ICUs) in 50 countries and found that hospital mortality was 34.9% for patients with mild ARDS, 40.3% for those with moderate ARDS, and 46.1% for those with severe ARDS [2]. A better understanding of the prognostic factors in ARDS is important for reducing mortality along with developing effective therapeutic strategies. As indicated in previous reports, the various risk factors for ARDS mortality include age, race and ethnicity, serious comorbidities such as HIV and malignancy, the ratio of partial pressure of arterial oxygen to fraction of inspired oxygen (PaO_2_/FiO_2_), and plateau pressure [3–7]. In addition, a report using the same sample as in the LUNG SAFE study found an association between per capita income and outcomes in ARDS [8]. Currently, Vietnam is one of the most dynamic emerging countries in East Asia. In 30 years, Vietnam has transformed from being one of the poorest countries in the world to becoming a lower middle-income country [9]. However, medical providers still have difficulties in caring for patients with ARDS in local settings owing to limited medical resources and a lack of advanced treatment strategies, such as extracorporeal membrane oxygenation, as well as physicians’ lower abilities to recognize ARDS in their patients. In addition, within the healthcare system in Vietnam, central tertiary hospitals are responsible for receiving patients who have difficulties being treated in local hospital settings [10, 11]. Therefore, the initiation of treatment in patients with ARDS is often delayed, including the administration of mechanical ventilation (MV) [12]. Under these conditions, the prognosis of ARDS in Vietnam might differ from that of other countries, regardless of income level. Understanding the country-specific etiologies and the disease risk and prognosis of ARDS are crucial for reducing mortality in Vietnam, as well as in other countries that face challenges in clinical practice owing to limited medical resources.

The aim of the present study was to elucidate the clinical epidemiology and disease prognosis in patients with ARDS in Vietnam. Our results can contribute to the reduction of ARDS mortality in Vietnam as well as in other countries, regardless of income level.

## Methods

### Study design and population

We carried out a retrospective observational study in National Bach Mai Hospital in Hanoi, Vietnam (hereafter, the central hospital), a tertiary hospital designated as the central hospital (level I) in northern Vietnam by the Ministry of Health (MOH) of Vietnam [10, 11]. In the healthcare system of Vietnam, central hospitals are responsible for educating hospital staff and treating patients who are unable to be adequately treated in local hospital settings, including provincial and district hospitals (levels II and III, according to the MOH Vietnam).

In this study, participants included adult patients (age ≥18 years) who were admitted to the Emergency Department or ICU of Bach Mai Hospital and diagnosed with ARDS from 2015 to 2017.

We collected data on patients’ general background, clinical condition, clinical time course, laboratory tests, radiographic findings, ventilator settings, gas exchange, and treatments. Data on laboratory tests, ventilator settings, and gas exchange were collected at two different time points: on days 1 and 3 after admission. The primary outcome of the study was hospital mortality, and a comparison was made between data of survivors and non-survivors and between data on days 1 and 3 after admission. Risk factors for mortality were assessed using logistic regression analysis.

The identification of ARDS was based on the diagnosis by the expert clinicians at the study site who treated the patients. The diagnosis and severity of ARDS were defined according to the Berlin definition of ARDS, which includes three stages of severity according to PaO_2_/FiO_2_ as follows: mild, 200<PaO_2_/FiO_2_≤300 mmHg; moderate, 100<PaO_2_/FiO_2_≤200 mmHg; and severe, PaO_2_/FiO_2_≤100 mmHg, under a minimum level of 5 cmH_2_O positive end-expiratory pressure (PEEP) [13, 14].

This study was approved by the Institutional Review Board of Bach Mai Hospital. Written informed consent was waived by the Board for this retrospective study, with public notification of the study made by public posting. Investigators kept the datasets in password-protected systems and presented data with the anonymity of study participants retained.

### Statistical analysis

Data are reported as percentages for categorical variables and as median with interquartile range (IQR: 25%–75%) or as mean with standard deviation (SD) for continuous variables. Comparisons were made between non-survivors and survivors and among severity of ARDS for each variable, using the χ^2^ test or Fisher’s exact test for categorical variables and Mann–Whitney *U* test, Kruskal–Wallis test, one-way analysis of variance, paired *t*-test, or Wilcoxon signed-rank test for continuous variables. Survival curves for duration of survival (in days) among patients with mild, moderate, and severe ARDS on day 1 and day 3 after admission and in patients transferred and those not transferred from local hospitals were analyzed using the Kaplan–Meier method; comparisons were made using the log-rank test.

Factors associated with mortality were estimated using a logistic regression analysis that included both independent variables of general characteristics as well as the baseline variables if the *p* value was <0.05 by univariate analysis between non-survivors and survivors. These variables included age; sex; comorbidities; treatment; length of hospitalization and MV at the local hospital; and the sequential organ failure assessment (SOFA) score, white blood cell count, and PaO_2_/FiO_2_ on days 1 and 3 after admission. A step-wise selection method was used to select variables and was conducted by the forced entry method.

Data were analyzed using IBM SPSS version 25.0 (IBM Corp., Armonk, NY, USA). For all analyses, significance levels were two-tailed, and *p*<0.05 was considered statistically significant.

## Results

### General characteristics, clinical time course, and outcomes of patients with ARDS in Vietnam according to survivability

During the observational period, a total 126 patients (male, 65.9%) were admitted and diagnosed with ARDS in the central hospital. Among them, 72 patients with ARDS died during hospitalization, and the mortality rate was 57.1%. The characteristics of patients were compared between non-survivors and survivors, as shown in Table 1.

**Table 1:** Characteristics of patients with ARDS: Comparison of survivors and non-survivors.

	Non-survivor n = 72	Survivor n = 54	*p* value
Gender–Male, n (%)	50 (69.4)	33 (61.1)	0.348
Age–median (IQR), yr.	56 (42–67)	47 (32–60)	0.027
< 20	3 (4.2)	3 (5.6)	
20–39	11 (15.3)	16 (29.6)	
40–59	28 (38.9)	21 (38.9)	
≥ 60	30 (41.7)	14 (25.9)	
Occupation, n (%)			0.618
Farmer	31 (43.1)	19 (35.2)	
Professionals (medical doctor, lawyer, teacher, etc.)	1 (1.4)	3 (5.6)	
House wife	3 (4.2)	1 (1.9)	
Employee	9 (12.5)	9 (16.7)	
Student	4 (5.6)	5 (9.3)	
Unemployment/Retired	24 (33.3)	17 (31.5)	
Smoking, n (%) (n = 118)			0.038
Current	19 (28.8)	16 (30.8)	
Former	13 (19.7)	2 (3.8)	
Never	34 (51.5)	34 (65.4)	
Comorbidities, n (%)			
Cerebral vascular disease	6 (8.3)	0 (0.0)	0.037
COPD	11 (15.3)	5 (9.3)	0.420
Chronic pulmonary diseases	3 (4.2)	5 (9.3)	0.287
Diabetes mellitus	11 (15.3)	7 (13.0)	0.801
Immunoincompetence	2 (2.8)	3 (5.6)	0.651
Chronic heart failure	6 (8.3)	1 (1.9)	0.237
Ulcer disease	1 (1.4)	2 (3.7)	0.576
Chronic renal failure	4 (5.6)	2 (3.7)	0.700
Chronic liver failure	11 (15.3)	6 (11.1)	0.603
Active neoplasm	1 (1.4)	2 (3.7)	0.576
Hematological disease	5 (6.9)	3 (5.6)	>0.999
others	14 (19.4)	8 (14.8)	0.637
Risk factor for ARDS, n (%)			
Pneumonia	68 (94.4)	49 (90.7)	0.496
Non-pulmonary sepsis	1 (1.4)	2 (3.7)	0.576
aspiration	0 (0.0)	4 (7.4)	0.032
Major trauma	0 (0.0)	0 (0.0)	-
Pulmonary contusion	3 (4.2)	1 (1.9)	0.635
Inhalation injury	10 (13.9)	8 (14.8)	>0.999
Severe burns	1 (1.4)	1 (1.9)	>0.999
Non-cardiogenic shock	10 (13.9)	8 (14.8)	>0.999
Drug overdose or addiction	7 (9.7)	5 (9.3)	>0.999
Pneumotoxic medication before ARDS onset, n (%)	8 (11.1)	6 (11.1)	>0.999

ARDS, acute respiratory distress syndrome; IQR, interquartile range; COPD, chronic obstructive pulmonary disease

The median age of survivors was less than that of non-survivors (*p* = 0.027), and 41.7% of non-survivors were aged ≥60 years. In most patients, ARDS was caused by pneumonia, with no significant difference between non-survivors (94.4%) and survivors (90.7%) (*p* = 0.496).

The clinical outcome and time course of patients with ARDS are shown in Table 2.

**Table 2:** Clinical outcome and time course of patients with ARDS: Comparison of survivors and non-survivors.

	Non-survivor n = 72	Survivor n = 54	*p* value
Severity of ARDS at admission*			0.163
Severe	42 (58.3)	25 (46.3)	
Moderate	26 (36.6)	21 (38.9)	
Mild	4 (5.6)	8 (14.8)	
**Outcomes**			
Died within 7 days from admission, n (%)	51 (70.8)	-	
Died within 28 days from admission, n (%)	71 (98.6)	-	
**Hospitalization in local prior to central**			
History of hospitalization in local	68 (91.9)	47 (90.4)	0.760
Length of hospitalization in local, median (IQR), d (n = 119)	8 (4–13)	17 (9–23)	<0.001
MV in local hospital (n = 119)	66 (93.0)	47 (87.0)	0.360
Length of MV in local, median (IQR), d (n = 113)	4 (2–8)	8 (4–14)	<0.001
**Clinical Time-course**			
Length of hospitalization in central, median (IQR), d	5 (3–9)	16 (11–20)	<0.001
Length of MV, median (IQR), d	5 (2–7)	9 (6–14)	<0.001

ARDS, acute respiratory distress syndrome; MV, mechanical ventilation; IQR, interquartile range

*Severity of ARDS was defined using the Berlin definition at the time of admission (diagnosed with ARDS), which is classified into three stages of severity according to the ratio of partial pressure of arterial oxygen to fraction of inspired oxygen (PaO_2_/FiO_2_): mild, 200<PaO_2_/FiO_2_≤300 mmHg; moderate, 100<PaO_2_/FiO_2_≤200 mmHg; and severe, PaO_2_/FiO_2_≤100 mmHg, under a minimum level of 5 cmH_2_O positive end-expiratory pressure [11, 12].

At the time of admission, the proportion of non-survivors (57.7%) who presented with severe ARDS was higher than that of survivors (46.3%). Among the total patients, 91.3% were transferred from local hospitals to the central hospital; the median length of stay in a local hospital was 8 days for non-survivors and 17 days for survivors (*p*<0.001). The duration of hospitalization (*p*<0.001) and MV (*p*<0.001) were significantly shorter in non-survivors than in survivors. A total 70.8% of non-survivors died within 7 days of admission to the central hospital, and all non-survivors died within 28 days of hospital admission, except one patient who was transferred from the Oncology Department within the Bach Mai Hospital and died 68 days after ICU admission.

Data regarding the chest radiograph images and laboratory findings among patients with ARDS according to survival are shown in Table 3.

**Table 3:** Chest radiograph imaging, severity of illness, and laboratory findings among patients with ARDS: Comparison of survivors and non-survivors.

	Non-survivor n = 72	Survivor n = 54	*p* value
**Chest imaging at admission, n (%)**			
Bilateral opacities (number of involved quadrants) (n = 114)			0.750
1/4	3 (4.7)	2 (4.0)	
2/4	2 (3.1)	0 (0.0)	
3/4	2 (3.1)	2 (4.0)	
4/4	57 (89.1)	46 (92.0)	
**Severity of illness on admission, mean (SD)**			
**1**^**st**^ **day of admission**			
SOFA (n = 111)	9.5 (3.4)	7.4 (3.4)	0.002
SAPS (n = 105)	47.8 (12.9)	36.6 (13.3)	<0.001
APACHE II (n = 122)	19.5 (6.8)	16.5 (6.0)	0.013
GCS (n = 114)	12.6 (6.1)	12.2 (2.4)	0.683
**3**^**rd**^ **day of admission**			
SOFA (n = 85)	11.7 (8.9)	7.4 (3.1)	0.004
SAPS (n = 82)	48.0 (15.1)	35.0 (11.5)	<0.001
APACHE II(n = 93)	20.3 (8.6)	15.3 (6.2)	0.002
GCS (n = 90)	9.9 (3.3)	12.1 (2.9)	0.001
**Laboratory findings, 1**^**st**^ **day of admission**, mean (SD)			
WBC	17.1 (16.0)	12.7 (7.0)	0.042
Platelet	189.6 (119.6)	169.4 (101.2)	0.307
Bilirubin (n = 80)	28.0 (33.4)	29.8 (56.5)	0.376
Creatinine	144.5 (146.2)	208.7 (561.9)	0.416
Albumin (n = 119)	25.1 (4.8)	27.5 (4.8)	0.010
**Laboratory findings, 3rd day of admission**, mean (SD)			
WBC (n = 95)	16.0 (9.8)	12.5 (5.4)	0.042
Platelet (n = 85)	182.2 (142.8)	146.5 (83.3)	0.147
Bilirubin (n = 80)	28.0 (33.4)	29.8 (56.5)	0.868
Creatinine (n = 95)	132.6 (90.3)	142.9 (183.4)	0.724
Albumin (n = 76)	25.9 (5.7)	28.1 (4.7)	0.072

ARDS: acute respiratory distress syndrome; SD, standard deviation; SOFA, sequential organ failure assessment; SAPS, simplified acute physiology score; APACHE, Acute Physiology and Chronic Health Evaluation; GCS, Glasgow Coma Scale; WBC, white blood cell count.

Although there was no significant difference, 89.1% of non-survivors and 92.0% of survivors presented bilateral opacities in four quadrants.

### Ventilator settings, gas exchange, and treatments in patients with ARDS

At the time of admission, over 80% of both non-survivors and survivors received volume-controlled ventilation (*p* = 0.797) (Table 4).

**Table 4:** Ventilator settings and gas exchange in patients with ARDS: Comparison of survivors and non-survivors.

	Non-survivor n = 72	Survivor n = 54	*p* value
**Ventilator mode**			
**1**^**st**^ **day of admission**			
VCV	52 (85.2)	40 (83.3)	0.797
others*	9 (14.8)	8 (16.7)	
**3**^**rd**^ **day of admission**			
VCV	30 (68.2)	32 (74.4)	0.637
Others*	14 (31.8)	11 (25.6)	
**Ventilator settings, mean (SD)**			
**1**^**st**^ **day of admission**			
FiO_2_, % (n = 123)	81.2 (23.4)	92.8 (60.6)	0.144
Set respiratory rate, 1/min (n = 116)	17.8 (4.9)	17.0 (4.4)	0.361
Tidal volume, mL/kg PBW (n = 108)	7.2 (1.3)	7.7 (1.1)	0.037
Set PEEP, cm H_2_O (n = 119)	9.3 (4.1)	10.4 (8.2)	0.376
**3**^**rd**^ **day of admission**			
FiO_2_, % (n = 96)	76.5 (22.2)	61.1 (20.9)	0.001
Set respiratory rate, 1/min (n = 90)	22.9 (6.6)	19.6 (7.1)	0.025
Tidal volume, mL/kg PBW (n = 72)	6.5 (1.1)	6.9 (2.0)	0.324
Set PEEP, cm H_2_O (n = 96)	12.0 (4.0)	10.1 (3.8)	0.018
**Gas exchange,** mean (SD)			
**1**^**st**^ **day of admission**			
PaO_2_/FiO_2_, mmHg	102.1 (52.5)	120.9 (66.7)	0.079
<150 mmHg–n (%)	56 (77.8)	37 (68.5)	0.307
PaO_2_	74.9 (31.1)	88.8 (41.8)	0.042
PaCO_2_	42.4 (15.5)	43.0 (11.2)	0.084
SpO_2_	88.1 (9.7)	90.7 (9.6)	0.142
pH	7.3 (0.2)	7.4 (0.1)	0.322
**3**^**rd**^ **day of admission** (n = 103)			
PaO_2_/FiO_2_, mmHg	126.3 (79.9)	183.9 (98.9)	0.002
<150 mmHg—n (%)	39 (76.5)	22 (42.3)	0.001
PaO_2_	84.9 (36.4)	100.0 (52.5)	0.095
PaCO_2_	50.8 (17.7)	41.8 (10.8)	0.002
SpO_2_	92.6 (6.1)	94.6 (8.1)	0.175
pH	7.3 (0.1)	7.4 (0.1)	< 0.001

*Others includes pressure control ventilation, controlled mechanical ventilation, pressure control ventilation, volume control plus, biphasic positive airway pressure.

ARDS, acute respiratory distress syndrome; VCV, volume control ventilation; PEEP, positive end-expiratory pressure; PaO_2_, partial pressure of arterial oxygen; FiO_2_, fraction of inspired oxygen; PaCO_2_, partial pressure of carbon dioxide; SpO_2_, oxygen saturation; SD, standard deviation.

**Fig 1:**
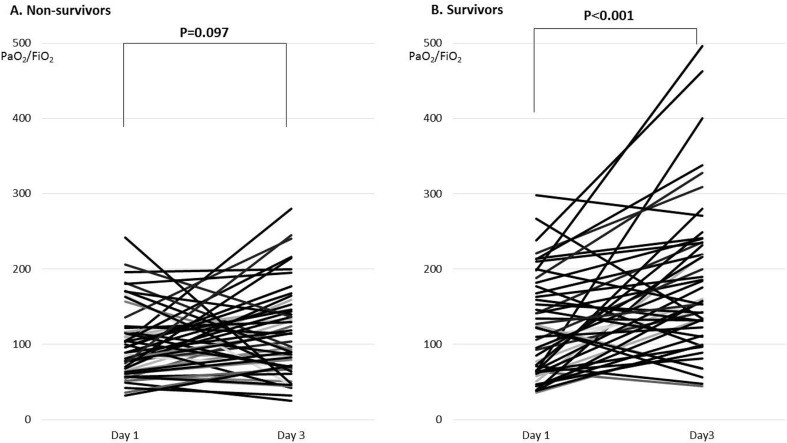
Difference in mean PaO_2_/FiO_2_ between day 1 and day 3 after admission in (A) non-survivors and (B) survivors.

In terms of ventilator settings, PEEP in survivors was maintained at ≥10 cmH_2_O on days 1 and 3; however, PEEP in non-survivors increased from 9.3 cmH_2_O on day 1 to 12.0 cmH_2_O on day 3 *(p* = 0.018) (Table 4). Regarding conditions of gas exchange, the mean (±SD) PaO_2_/FiO_2_ between non-survivors (102.1±52.5 mmHg) and survivors (120.0±66.7 mmHg) on day 1 was not significantly different (*p* = 0.079), nor was the frequency of patients with a PaO_2_/FiO_2_ of <150 mmHg (*p =* 0.307). On day 3 after admission, the mean (±SD) PaO_2_/FiO_2_ between non-survivors (126.3±79.9 mmHg) and survivors (183.9±98.9 mmHg) was significantly different (*p* = 0.002), as was the frequency of patients with a PaO_2_/FiO_2_ of <150 mmHg (*p =* 0.001). The difference in the mean PaO_2_/FiO_2_ between day 1 and day 3 after admission in non-survivors was not significant (*p* = 0.097) (Fig 1A), while the mean PaO_2_/FiO_2_ in survivors was significantly increased (*p*<0.001) (Fig 1B).

Treatments in patients with ARDS are shown in Table 5.

**Table 5:** Treatment of ARDS: Comparison of non-survivors and survivors.

	Non-survivor n = 72	Survivor n = 54	*p* value
Sedation	70 (97.2)	51 (94.4)	0.650
Neuromuscular blocker	43 (59.7)	31 (58.5)	0.518
Systemic corticosteroids	4 (5.6)	6 (11.3)	0.200
Antibiotics	72 (100.0)	54 (100.0)	-
Antifungal	5 (6.9)	5 (9.4)	0.742
Antiviral	11 (15.3)	21 (39.6)	0.003
Prone positioning	15 (20.8)	8 (15.1)	0.488
ECMO	3 (4.2)	7 (13.2)	0.095
CHDF	22 (30.6)	24 (46.2)	0.091
HFOV	6 (8.3)	5 (9.6)	>0.999
Recruitment maneuvers	8 (11.1)	14 (26.9)	0.032
Tracheostomy	0 (0.0)	6 (11.3)	0.005

ARDS: acute respiratory distress syndrome; ECMO, extracorporeal membrane oxygenation; CHDF, continuous hemodiafiltration; HFOV, high-frequency oscillation ventilation.

Among all patients, antiviral drugs were administered more frequently to survivors than non-survivors (*p* = 0.003). Recruitment maneuvers were applied more often to survivors than to non-survivors (*p* = 0.032). Although the number of patients was small, only survivors underwent tracheostomy (*p* = 0.005).

### Outcome and disease prognosis of patients with ARDS as classified by ARDS severity according to the Berlin definition

The outcome and disease prognosis of patients with ARDS were compared by the severity category of ARDS according to the Berlin definition, as shown in Table 6.

**Table 6:** Outcomes and disease prognosis of patients with ARDS according to severity category.

	mild n = 12	moderate n = 47	Severe n = 67	*p* value
**General characteristics**				
Gender–Male, n (%)	9 (75.0)	27 (57.4)	47 (70.1)	0.118
Age–median (IQR), yr.	54 (40)	54 (31–69)	50 (41–61)	0.873
< 20	0 (0.0)	3 (6.4)	3 (4.5)	0.016
20–39	3 (25.0)	13 (27.7)	11 (16.4)	
40–59	4 (33.3)	12 (25.5)	33 (49.3)	
≥ 60	5 (41.7)	19 (40.4)	20 (29.9)	
Transferred from local hospital, n (%)	11 (91.7)	44 (93.6)	60 (89.6)	0.681
**Mortality**				
7 days mortality (n = 97)	2 (16.7))	18 (38.3)	31 (46.3)	0.148
28 days mortality	4 (33.3)	27 (57.4)	42 (62.7)	0.176
Hospital mortality	4 (33.3)	27 (57.4)	43 (64.2)	0.142
**Length of hospital stay**–median (IQR), days.				
All patients	9 (3–20)	9 (5–18)	8 (3–15)	0.552
Surviving patients	18 (4–23)	15 (11–25)	16 (12–21)	0.918
Non-surviving patients	5 (1–9)	5 (4–10)	4 (3–9)	0.420
**Length of stay in local hospital** (n = 119)—mean (SD), days	12.7 (12.0)	12.3 (9.6)	11.3 (11.7)	0.929
**Length of MV days in local hospital** (n = 117)–mean (SD), days	10.5 (9.5)	8.2 (7.5)	6.9 (7.2)	0.307
**Severity of ARDS on day 3 by Berlin definition** (n = 95)				0.023
Mild	6 (66.7)	8 (20.0)	8 (14.8)	
Moderate	2 (22.2)	20 (50.0)	23 (42.6)	
Severe	1 (11.1)	12 (30.0)	23 (42.6)	
**Progression/improvement of ARDS severity on day3**—n (%)				
Progression to moderate	2 (1.6)	-	-	
Progression to severe	1 (0.8)	-	-	
Progression to severe	-	12 (9.5)	-	
Improvement to mild	-	8 (6.3)	-	
Improvement to moderate	-	-	23 (18.3)	
Improvement to mild	-	-	7 (5.6)	

ARDS, acute respiratory distress syndrome; IQR, interquartile range; SD, standard deviation; MV, mechanical ventilation

Overall hospital mortality was 58.7%. There was no significant difference in 7- or 28-day mortality among the ARDS severity groups. Although the number days of hospital stay was not significant among the ARDS severity groups, the median length of hospital stay in non-survivors was shorter than that in survivors. Although statistical significance was not observed, the mean number of days of MV in the local hospital was the longest in patients with mild ARDS among all ARDS severities.

The number of patients with mild, moderate, and severe ARDS on day 3 after admission was 16 (16.8%), 45 (47.4%), and 34 (35.8%), respectively. Between day 1 and day 3 after admission, the severity of ARDS improved in 41 (32.5%) patients with moderate and severe ARDS and worsened in 15 (11.9%) patients with mild or moderate ARDS (Table 6).

**Fig 2:**
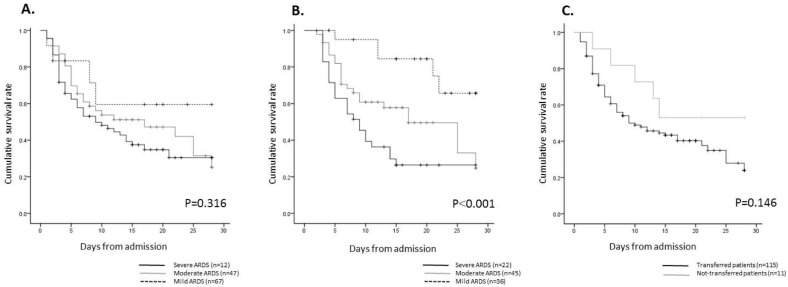
**Kaplan–Meier curves of probability of hospital mortality according to ARDS severity on (A) day 1 or (B) day 3 after admission, and patients (C) transferred or not transferred from a local hospital.** The log-rank test was used to assess differences in the curves.

The Kaplan–Meier curves revealed significant differences in the probability of hospital mortality among the three ARDS severity groups on day 3 (Fig 2B); there were no significant differences among the groups on day 1 (Fig 2A). In addition, no significant differences were observed between patients who were transferred and those who were not transferred from a local hospital (Fig 2C).

### Factors related to survival of patients with ARDS in Vietnam

A multivariate logistic regression analysis revealed that PaO_2_/FiO_2_ on day 3 after admission [odds ratio (OR), 1.010; 95% confidence interval (CI), 1.003–1.017] and length of stay in a local hospital (OR, 1.099; 95% CI, 1.028–1.176) were factors related to the survival of patients with ARDS in Vietnam (Table 7). The SOFA score on day 1 of admission also tended to be related to the patients’ survival (OR, 0.842; 95% CI, 0.708–1.002).

**Table 7:** Factors related to survival of patients with ARDS.

	coefficient	SE	*p* value	OR	95% CI
constant	-1.495	1.047			
PaO_2_/FiO_2_ on day3 from admission	0.010	0.004	0.007	1.010	1.003–1.017
Length of hospitalization in local	0.095	0.034	0.006	1.099	1.028–1.176
SOFA on Day 1	-0.172	0.088	0.052	0.842	0.708–1.002

ARDS, acute respiratory distress syndrome; SE, standard error; OR, odds ratio; 95% CI, 95% confidence interval; PaO_2_/FiO_2_, ratio of partial pressure of arterial oxygen to fraction of inspired oxygen; SOFA, sequential organ failure assessment.

## Discussion

The present study revealed that mortality in patients with ARDS in Vietnam was high at 57.1%. Among all patients, 91.3% were transferred from local hospitals with severe pneumonia and diagnosed with ARDS at the central hospital. Higher PaO_2_/FiO_2_ on the third day after hospital admission and longer stay in a local hospital, which may have been related to mild disease severity, were related to better survival.

A unique characteristic of patients with ARDS in Vietnam is that many of these patients are diagnosed with severe pneumonia in local hospitals and are then transferred to a central hospital if their conditions become severe. This leads to delayed diagnosis and delayed initiation of treatment for ARDS, which can in turn lead to high mortality. However, transferring patients with severe ARDS is based on the framework within the management system of healthcare provision laid out by the MOH of Vietnam known as the Direction of Healthcare Activities (DOHA) [10, 11]. In this system, healthcare facilities are divided into four levels according to administrative structure: central (level I), provincial (level II), district (level III), and community (level IV) [10]. The DOHA system requires health facilities at higher administrative levels to support those at lower levels, enabling them to deliver medical services to local communities as well as to improve the quality of medical services in Vietnam [10, 11]. Bach Mai Hospital, the site of this study, is the central hospital (level I) in northern Vietnam and is responsible for treating patients with severe conditions from lower level hospitals as well as educating and training medical providers at local hospitals. This is one of the main reasons for the present study; namely, to examine the outcome of patients transferred from local hospitals. This situation is related to the high number of patients with severe ARDS at the time of admission. Although early transfer to a central hospital and early initiation of treatment for ARDS are crucial for patient survival, the present study findings indicated that a longer stay in a local hospital is related to better survival. This means that if the condition of a patient is less severe, clinicians in local hospitals keep patients in their hospital and provide medical care to these patients without transferring them to a higher-level facility. Therefore, we found greater severity of illness with shorter length of stay in a local hospital. As a result, the duration of MV in patients with mild ARDS at local hospitals was longer than that in patients with moderate and severe ARDS. The clinicians in local hospitals continue providing care for patients in their hospitals if the patients’ conditions remain stable. However, if the length of MV becomes long or the patients’ condition worsens, the clinicians send the patients to the central hospital. This can also explain the low incidence of mild ARDS at the time of admission. A longer stay in a local hospital may also be related to clinicians’ lack of knowledge and low recognition of ARDS at these hospitals, as well as the lower quality of treatment and management of MV in lower-level facilities. Our previous survey in Vietnam indicated that clinicians in local hospitals would like to consult with an expert in a central hospital regarding patients with ARDS [15]. Thus, strengthening education for medical providers in local hospitals and providing a system for clinicians in lower-level facilities to consult with experts at a central hospital could lead to more timely transfer of critically ill patients from a local to a central hospital.

The poor recognition of ARDS may also be related to a lack of medical resources in local settings. For instance, even at provincial level, medical equipment such as portable chest radiograph and computed tomography devices are not available in all hospitals. In addition, transferring patients with severe pneumonia from a local to a central hospital may result in worsening of their critical condition. There is a high possibility that patients could be transferred with no intubation, ventilation, PEEP, and so on. There are limited data in terms of the conditions during transfer of patients in Vietnam; however, a previous survey indicated that there are many risk factors for higher numbers of patient transfers and poor-quality pre-hospital care [16]. The local hospitals under the umbrella of Bach Mai Hospital are distributed across northern Vietnam. The conditions for transport by car over long distances and many hours are critical for patients with severe pneumonia and may affect the severity and progression of their disease. Moreover, the SOFA score at the time of admission was a predictive factor for the survivability of patients, while the SOFA score on day 3 did not predict patients’ survival. A study in Cambodia reported that patients with avian influenza H5N1 virus infection traveled up to 476 km prior to hospital admission [17]. Thus, along with socioeconomic conditions [18], the condition of patient transport could be a major risk factor related to disease severity and prognosis in low- and middle-income countries. Although we found no statistically significant differences between patients who were transferred from local hospitals and those who were not, Kaplan–Meier curves revealed that transferred patients tended to have shorter survival times than non-transferred patients. Detailed examination is necessary to confirm this result using a larger sample size.

After being admitted to the central hospital, PaO_2_/FiO_2_ on day 3 after admission was also considered to be a factor influencing patient survival. Although oxygenation in survivors tended to be lower than that in non-survivors, the mean PaO_2_/FiO_2_ on day 1 after admission was not significantly different between non-survivors and survivors. However, the mean PaO_2_/FiO_2_ on day 3 after admission differed significantly between non-survivors and survivors. These results suggest that improvement of oxygenation within 3 days of hospitalization had a substantial influence on the survival of patients with ARDS, even those with severe ARDS, at the time of admission. This result is compatible with that of a previous study, in which the multivariate analysis showed that PaO_2_/FiO_2_ on the third day after ARDS diagnosis is strongly associated with ARDS-associated mortality [19]. Diffuse alveolar damage (DAD) has been described as the pathological hallmark of ARDS [20, 21]. An autopsy study evaluating the accuracy of the Berlin definition indicated a correlation of DAD with ARDS severity [21]. That report also described that the presence of DAD was markedly higher in patients who met these clinical criteria for ARDS for more than 72 hours than in those who met the criteria for less than 72 hours; the incidence of DAD showed a difference after 72 hours [21]. Another study indicated that PaO_2_/FiO_2_ on the third day after diagnosis was a predictive factor for ARDS mortality [22]. Moreover, in our study, the PEEP level on day 3 of hospitalization was different between non-survivors and survivors; PEEP levels on day 1 showed no difference. A previous study indicated that the pulmonary and extrapulmonary origin of ARDS may influence the response to PEEP [23, 24]. However, other studies showed that the origin of ARDS has no impact on the alveolar damage induced by PEEP or on mortality [22–24]. Under these controversial findings, the results of our multivariate analysis did not identify PEEP as an influencing factor for mortality.

The etiology of ARDS varies, and some types of ARDS have rapid progression depending on their cause [12, 25]. Detailed examination of the Vietnamese-specific etiology of ARDS, such as avian influenza virus infection, is required for further consideration of the clinical management of ARDS in Vietnam.

The limitations of the present study were associated with its retrospective design. The causality between risk factors and death was not able to be proven. Because there are various levels of medical facilities in Vietnam, the study was conducted in a single hospital. This hospital is the highest-level medical facility owned by the MOH of Vietnam, which helped to unify the quality of care and diagnosis of patients with ARDS in the present study. However, it resulted in a small sample size for a clinical epidemiological study. Most patients in this study were transferred from the local hospital with severe pneumonia, and we relied on the clinical diagnosis for the identification of ARDS at the study site. Therefore, some of the patients might have already developed ARDS in the local hospital but received a diagnosis of ARDS after admission to the central hospital. This may have also caused the low incidence of mild ARDS and non-pulmonary ARDS in the present study. Moreover, were unable to evaluate treatments in relation to mortality among our patients with ARDS. However, to the best of our knowledge, this is the first study to evaluate the clinical epidemiology and mortality risk in adult patients with ARDS in Vietnam. These results will provide important information to physicians and can help to reduce mortality among patients with ARDS in Vietnam and other low- and middle-income countries.

## Conclusions

Patients with severe ARDS were admitted to the central tertiary hospital in northern Vietnam with high mortality. Improvements in human, medical, and sociological resources in local will contribute to reducing the mortality of ARDS in Vietnam.

## Supporting information

S1 Table (XLSX)
